# Diversified Effects of COVID-19 as a Consequence of the Differential Metabolism of Phospholipids and Lipid Peroxidation Evaluated in the Plasma of Survivors and Deceased Patients upon Admission to the Hospital

**DOI:** 10.3390/ijms231911810

**Published:** 2022-10-05

**Authors:** Neven Žarković, Wojciech Łuczaj, Iwona Jarocka-Karpowicz, Biserka Orehovec, Bruno Baršić, Marko Tarle, Marta Kmet, Ivica Lukšić, Michał Biernacki, Elżbieta Skrzydlewska

**Affiliations:** 1 Laboratory for Oxidative Stress (LabOS), Ruđer Bošković Institute, HR-10000 Zagreb, Croatia; 2 Department of Analytical Chemistry, Medical University of Bialystok, A. Mickiewicza 2D, 15-222 Bialystok, Poland; 3 Clinical Hospital Dubrava, HR-10000 Zagreb, Croatia; 4 Medical Faculty, University of Zagreb, HR-10000 Zagreb, Croatia

**Keywords:** COVID-19, SARS-CoV-2, plasma, survived patients, deceased patients, endocannabinoids, eicosanoids, oxidative stress, lipid peroxidation, inflammation

## Abstract

As a result of SARS-CoV-2 infection, inflammation develops, which promotes oxidative stress, leading to modification of phospholipid metabolism. Therefore, the aim of this study is to compare the effects of COVID-19 on the levels of phospholipid and free polyunsaturated fatty acids (PUFAs) and their metabolites produced in response to reactions with reactive oxygen species (ROS) and enzymes (cyclooxygenases-(COXs) and lipoxygenase-(LOX)) in the plasma of patients who either recovered or passed away within a week of hospitalization. In the plasma of COVID-19 patients, especially of the survivors, the actions of ROS and phospholipase A2 (PLA2) cause a decrease in phospholipid fatty acids level and an increase in free fatty acids (especially arachidonic acid) despite increased COXs and LOX activity. This is accompanied by an increased level in lipid peroxidation products (malondialdehyde and 8-isoprostaglandin F2α) and lipid mediators generated by enzymes. There is also an increase in eicosanoids, both pro-inflammatory as follows: thromboxane B2 and prostaglandin E2, and anti-inflammatory as follows: 15-deoxy-Δ-12,14-prostaglandin J2 and 12-hydroxyeicosatetraenoic acid, as well as endocannabinoids (anandamide-(AEA) and 2-arachidonylglycerol-(2-AG)) observed in the plasma of patients who recovered. Moreover, the expression of tumor necrosis factor α and interleukins (IL-6 and IL-10) is increased in patients who recovered. However, in the group of patients who died, elevated levels of N-oleoylethanolamine and N-palmitoylethanolamine are found. Since lipid mediators may have different functions depending on the onset of pathophysiological processes, a stronger pro-inflammatory response in patients who have recovered may be the result of the defensive response to SARS-CoV-2 in survivors associated with specific changes in the phospholipid metabolism, which could also be considered a prognostic factor.

## 1. Introduction

COVID-19, caused by severe acute respiratory syndrome coronavirus 2 (SARS-CoV-2) initiates an abnormal immune response and is characterized by a wide spectrum of clinical manifestations [1], which due to the high risk of developing sepsis, acute respiratory distress syndrome (ARDS), heart failure shock or shock is associated with increased mortality in the human population [2]. Metabolic studies indicate that the development of COVID-19 accompanied by increased ROS generation promotes oxidative stress and inflammation, leading to severe lung as well as other organ damage [3]. Oxidative stress contributes significantly to the pathogenesis of viral infections by promoting both viral replication and monocyte activation, which participate in the immune response, remove pathogens by phagocytosis, as well as participate in inflammatory processes [4]. Some consequences of oxidative stress in COVID-19 patients have been demonstrated earlier [5,6]. 

Changes in phospholipid metabolism are both a direct result of the action of reactive oxygen species (ROS) on both free and phospholipid fatty acids, as well as increased lipid metabolism dependent on enzymes such as phospholipase A2 (PLA2), cyclooxygenase (COX), and lipoxygenase (LOX) action [5,7]. Elevated levels of 4-hydroxynonenal (4-HNE) and malondialdehyde (MDA) were observed in the plasma of patients with COVID-19 [8]. Of particular interest, we found 4-HNE, which was increased in the blood of COVID-19 patients and was found abundant in the vital organs of the deceased patients [6,9]. Accordingly, we assume that inflammation, together with oxidative stress and altered lipid metabolism, might be important parameters of the aggressive SARS-CoV-2 infection and systemic reaction to it. 

Increased activity of lipid metabolizing enzymes results in the enhanced generation of lipid mediators from the group of both endocannabinoids and eicosanoids, which are involved in modifications of both oxidative stress and inflammation, mainly through activation of G protein-coupled receptors [5,10]. Numerous experimental studies indicate that activation of CB1 receptors is crucial for the development of an effective innate immune response during inflammation, while activation of CB2 receptors prevents further damage caused by inflammation, e.g., immunosuppression during sepsis [10,11]. Activation of the endocannabinoid system may also reduce inflammation and thus prevent damage to sepsis-sensitive organs often associated with SARS-CoV-2 virus infections [10,12]. So far, however, there is a lack of experimental evidence showing the direct effect of COVID-19 on modifications of the endocannabinoid system and the potential consequences of such changes. The increase in PLA2 activity as a result of oxidative stress [13] promotes the release of polyunsaturated fatty acids (PUFA), which are substrates for COX and LOX, generating further groups of lipid mediators, including eicosanoids [7]. The emerging eicosanoids play an important role in both the initiation of inflammatory reactions and because they are involved in the process of terminating acute inflammation and returning to homeostasis [14]. Especially well understood is the metabolism of arachidonic acid, which is a precursor of pro-inflammatory prostaglandins, leukotrienes, and thromboxanes as well as anti-inflammatory lipoxins [14]. These compounds play a crucial role in regulating viral replication as well as modifying host innate and adaptive immune responses [15,16,17]. Among eicosanoids, prostoglandins and leukotrienes play a significant role in activating the immune response [18]. Excessive production of these molecules is associated with the propagation of both local and systemic inflammation through the dysregulation of the vascular response and leukocyte activation and recruitment [19]. While leukotrienes show ionotropic effects and promote smooth muscle contraction in the lungs, prostaglandins show immunosuppressive effects by inhibiting effector T-cell responses, an important mechanism in SARS-CoV infections [20]. On the other hand, lipid mediators such as lipoxins exhibit strong immune-regulatory effects during viral infections [21]. Specialized pro-resolving mediators have been found to reduce the production of pro-inflammatory cytokines such as IL-6 and IL-1β, which are involved in the development of COVID-19 [22,23]. However, the overproduction of eicosanoids is directly related to the uncontrolled and rapid generation and self-reinforcing activation of the cytokine cascade, including pro-inflammatory ones such as IFN-γ, IL-6, and TNFα [24], the synthesis of which is inhibited by IL-10 [25]. 

Consequently, since lipids are not only the main components of membrane structures but also a source of energy, while lipid mediators play an important role in intercellular signaling, viruses use and modify lipid metabolism to promote viral replication [26]. Therefore, the aim of the present study was to compare the effects of SARS-CoV-2 infection on the levels of lipid mediators belonging to endocannabinoids and eicosanoids generated from PUFAs in enzyme-dependent reactions and lipid peroxidation products generated during ROS reactions with phospholipid/free PUFAs in the plasma of patients, both those who recovered and those who died.

## 2. Results 

Results of blood laboratory tests of healthy subjects and patients with COVID-19 are outlined in Table 1 Among the examined biochemical parameters, we found significantly higher levels of IL-6 in the plasma of deceased COVID-19 patients in comparison to recovered patients.

**Figure 1:**
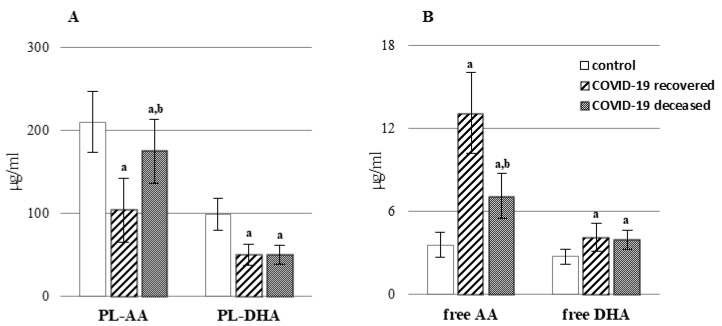
The levels of phospholipid (**A**) and free fatty acids (**B**) in the plasma of patients with COVID-19, including those who recovered (n = 66), and those who deceased (n = 22) as well as healthy subjects (n = 33). Phospholipid arachidonic acid (PL-AA), phospholipid docosahexaenoic acid (PL-DHA). Data points represent the mean ± SD; a, significantly different from healthy subject, *p* < 0.05; b, significantly different from patients with COVID-19 recovered, *p* < 0.05.

**Figure 2:**
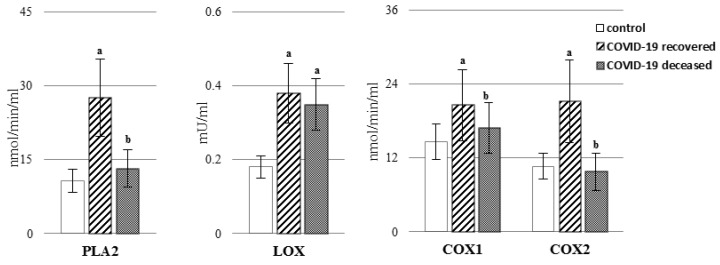
The activity of phospholipase A2 (PLA_2_), lipoxygenase (LOX), and cyclooxygenase-1/2 (COX-1/2) in the plasma of patients with COVID-19, including those who recovered (n = 66) and those who deceased (n = 22) as well as healthy subjects (n = 33). Data points represent the mean ± SD; a, significantly different from healthy subject, *p* < 0.05; b, significantly different from patients with COVID-19 recovered, *p* < 0.05.

COVID-19 promoted disorders of plasma phospholipid metabolism such as lipid peroxidation and the action of enzymes (PLA2, COXs, and LOXs) which affect the metabolism of fatty acids (Figure 1 and Figure 2, Supplementary Table S1). The increase in activity of PLA2 in plasma, especially in patients who recovered, was observed (Figure 2, Supplementary Table S1) and led to the release of fatty acids from the phospholipid structure. Consequently, it resulted in a decreased level of phospholipid AA and DHA acids (Figure 1, Supplementary Table S1). There is no doubt that the most significant changes in the level of fatty acids, especially AA, were observed in patients who recovered. In the case of docosahexaenoic acid, both groups of patients show similar dependencies. At the same time, the above-mentioned changes were accompanied by an increase in the level of the free fatty acids in the plasma of COVID-19 patients. A particularly high increase (approx. 3-fold) of AA acid in the plasma of patients who recovered was observed. As in the case of PLA2, LOX activity was increased in the plasma of COVID-19 patients, and for both enzymes, higher activity was observed in patients who survived (Figure 2, Supplementary Table S1). Similar dependencies were found in relation to the activity of two distinct isoforms of cyclooxygenase—COX1 (constitutive, responsible for the production of prostaglandins associated with physiological function) and COX2 (inducible, induced as a result of inflammation and responsible for producing prostaglandins such as PGE2).

**Figure 3:**
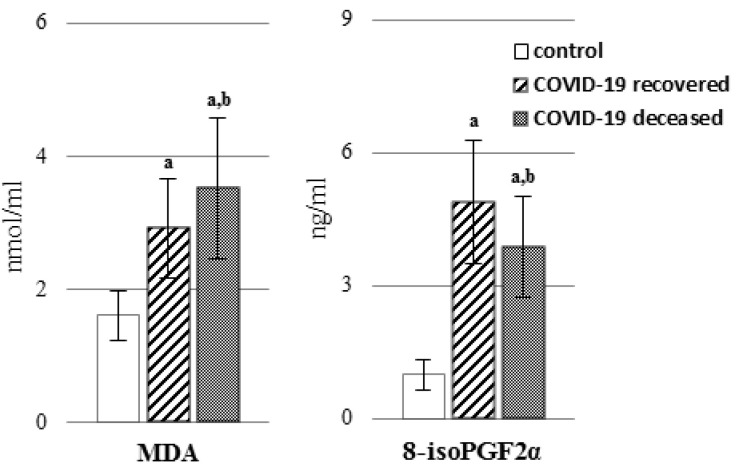
The levels of lipid peroxidation products [MDA, 8-isoPGF_2α_] in the plasma of patients with COVID-19, including those who recovered (n = 66), and those who deceased (n = 22) as well as healthy subjects (n = 33). Data points represent the mean ± SD; a, significantly different from healthy subject, *p* < 0.05; b, significantly different from patients with COVID-19 recovered, *p* < 0.05.

As a result of oxidative stress and increased ROS-dependent reactions as well as PLA2 action, increased lipid peroxidation occurred in the plasma of COVID-19 patients. This has been confirmed by increased levels of the oxidative fragmentation products of mainly arachidonic acid, such as MDA, and oxidative cyclization products of phospholipids, such as 8-isoPGF2α (Figure 3, Supplementary Table S1). At the same time, patients who died as a result of COVID-19 tended to have a more severe increase in MDA levels and a lesser increase in the 8-isoPGF2α level.

**Figure 4:**
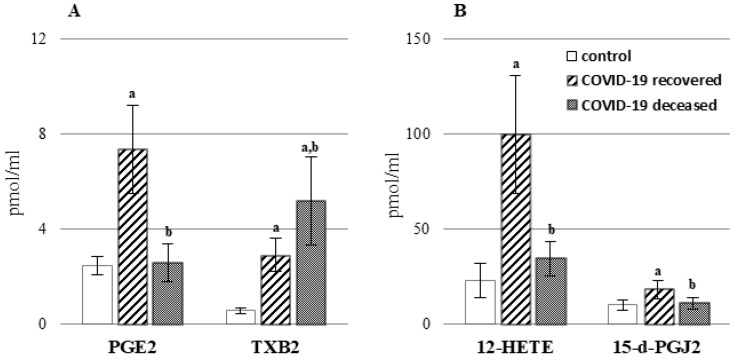
The level of pro-inflammatory eicosanoids (**A**): [thromboxane B2 (TXB_2_) and prostaglandin E2 (PGE_2_)] and anti-inflammatory eicosanoids (**B**): [15-deoxy-delta12,14-prostaglandin J2 (15d-PGJ2) and 12-hydroxyeicosatetraenoic acid (12-HETE)] in the plasma of patients with COVID-19, including those who recovered (n = 66) and those who deceased (n = 22) as well as healthy subjects (n = 33). Data points represent the mean ± SD; a, significantly different from healthy subject, *p* < 0.05; b, significantly different from patients with COVID-19 recovered, *p* < 0.05.

An increase in the level of polyunsaturated free fatty acids and the activity of enzymes responsible for the biosynthesis of eicosanoids (COXs and LOX) may result in an increase in the level of lipid mediators, which include eicosanoids (Figure 4, Supplementary Table S1). The levels of both the pro-inflammatory eicosanoids TXB2 and PGE2 and the anti-inflammatory eicosanoids 15d-PGJ2 and 12-HETE were found to be elevated in the plasma of COVID-19 survivors. In contrast, only the level of thromboxane B2 (TXB2) was elevated in the plasma of patients who died as a result of COVID-19.

**Figure 5:**
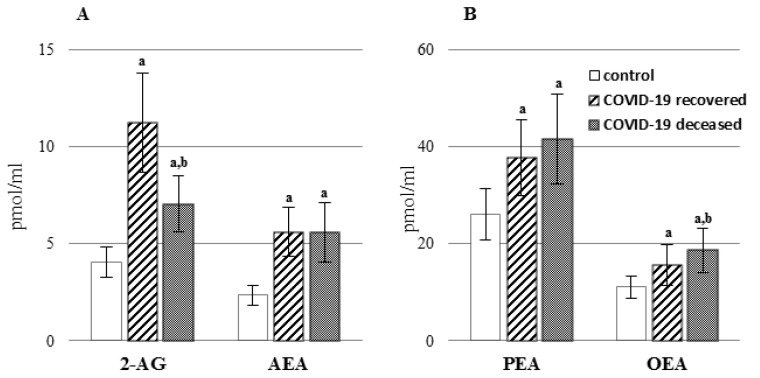
The levels of endocannabinoids (**A**): [N-arachidonoylethanolamine (AEA), 2-arachidonoylglycerol (2-AG)] and other bioactive acylethanolamides (**B**): [*N*-oleoylethanolamine (OEA), *N*-palmitoylethanolamine (PEA)] in the plasma of patients with COVID-19, including those who recovered (n = 66) and those who deceased (n = 22) as well as healthy subjects (n = 33). Data points represent the mean ± SD; a, significantly different from healthy subject, *p* < 0.05; b, significantly different from patients with COVID-19 recovered, *p* < 0.05.

In this study, we also assessed the levels of lipid mediators belonging to the group of endocannabinoids (Figure 5, Supplementary Table S1), which are lipid hormones found in all organs and body fluids that control a wide range of physiological functions. The two most studied endocannabinoids are the arachidonic acid derivatives, N-arachidonoylethanolamine (AEA) and 2-arachidonoyl glycerol (2-AG). Moreover, other mimic compounds derived from other fatty acids (palmitic and oleic) perform important functions in the body. These include palmitoylethanolamide (PEA) and oleoylethanolamide (OEA). Increased levels of both classic endocannabinoids (AEA and 2-AG), as well as two mimic compounds (PEA and OEA), have been demonstrated in the plasma of both survivors of the infection and deceased patients. 

**Figure 6:**
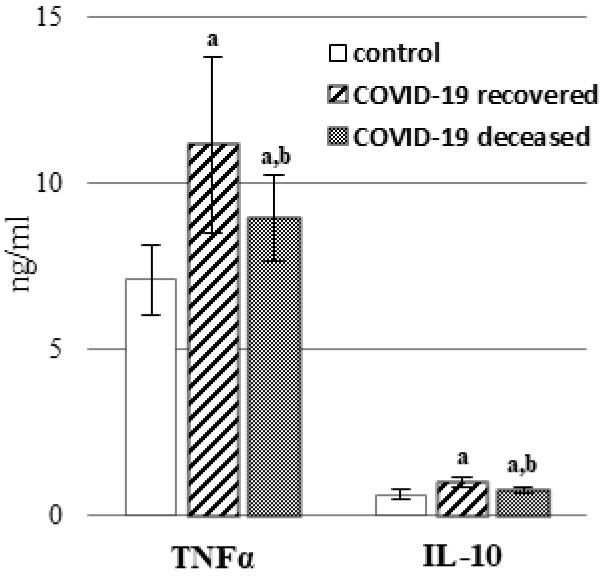
The level of anti-inflammatory interleukin 10 (IL-10) and pro-inflammatory tumor necrosis factor alpha (TNF-α) in the plasma of patients with COVID-19, including those who recovered (n = 66) and those who deceased (n = 22) as well as healthy subjects (n = 33). Data points represent the mean ± SD; a—significantly different from healthy subjects, *p* < 0.05; b—significantly different from recovered patients with COVID-19, *p* < 0.05.

Oxidative stress is usually accompanied by inflammation, and this also applies to COVID-19 patients. The results of the present study showed that in the plasma of COVID-19 patients, the expression of pro-inflammatory cytokines—TNFα (Figure 6, Supplementary Table S1) and IL-6 (Table 1) and the anti-inflammatory interleukin—IL-10 (Figure 6, Supplementary Table S1) increases, especially in the plasma of recovered COVID-19 patients. 

## 3. Discussion

It is known that SARS-CoV-2 infection leads to inflammation, which promotes the development of oxidative stress, which in turn may contribute to increased inflammation [4]. On the other hand, under conditions of oxidative stress, elevated levels of ROS promote oxidative modifications of biologically relevant components of the body, including macromolecular compounds such as lipids, proteins, and nucleic acids [4]. In addition, in the early phase of the host response to pathogens, polyunsaturated fatty acids are released from phospholipids, especially from blood cells such as neutrophils, dendritic cells, and macrophages [27]. The results of the present study indicate decreased levels of phospholipid PUFAs, such as arachidonic and docosahexaenoic acids, which, in the context of elevated levels of 8-isoPGF2α, and MDA (Supplementary Figure S1), are products of lipid peroxidation [28]. Elevated levels of MDA are observed in the plasma of all COVID-19 patients. These findings support the earlier view that there is a significant correlation between lipid markers of oxidative stress and respiratory viral infections, especially those caused by respiratory viruses [29]. Therefore, in order to improve the effectiveness of treatment in relation to COVID-19 patients, pharmacotherapy supported by PUFAs may be suggested. 

Lipid mediators play an important role as intercellular signaling factors; therefore, viruses harness and modify both lipid signaling and their metabolism to promote viral replication [26]. As a result of the enzymatic metabolism of phospholipids, their metabolites from the group of endocannabinoids are generated (Supplementary Figure S1). The two most common, and therefore the best-studied endocannabinoids, are arachidonic acid derivatives such as N-arachidonoyl ethanolamine (AEA) and 2-arachidonoylglycerol (2-AG), while derivatives of other fatty acids (palmitic and oleic acids) are endocannabinoid-related compounds such as N-oleoylethanolamine (OEA), N-palmitoylethanolamine (PEA) [30,31]. AEA and 2-AG levels were significantly elevated primarily in patients who survived, while the levels of PEA and OEA were increased, especially in patients who died. It is known that AEA can attenuate ARDS through the activation of anti-inflammatory pathways in immunosuppressive cells such as myeloid-derived suppressor cells (MDSCs) and regulatory T cells [12]. Additionally, in an experiment on mice with ARDS, it was shown that AEA introduced into experimental therapy significantly decreased the expression of pro-inflammatory IL-6 [32], an elevated level of which was observed in this study, especially in patients who did not survive. Moreover, in another study, both in blood serum and bronchoalveolar lavage fluid, a similar relationship was detected, especially in patients with respiratory tract infections, including patients with COVID-19 [33,34]. In addition, AEA and 2-AG have been shown to suppress pro-inflammatory cytokines and increase anti-inflammatory cytokines in other viral infections such as human immunodeficiency virus (HIV) or Theiler virus [35,36,37]. Since AEA and 2-AG are cannabinoid receptors (CB1 and CB2) agonists [30], so activation of the CB2 receptors, which mainly occur in immune cells [38], promotes TNFα downregulation and inhibition of leukocyte recruitment, which reduces inflammation in various diseases [38]. We also observed the activation of CB2 receptors in the granulocytes of patients with COVID-19 (data not yet published). In the present study, especially in deceased patients, the levels of mimic endocannabinoids such as PEA and OEA were elevated in plasma. Both compounds exhibit antioxidant and anti-inflammatory properties, thus preventing endothelial damage [39,40], but their high levels did not prevent the severe and fatal course of COVID-19. 

Oxidative stress and increased levels of pro-inflammatory cytokines promote the activation of lipolytic enzymes, especially phospholipase A2 [41], which is also observed in the plasma of COVID-19 patients. The intensity of the phospholipid hydrolysis is evidenced by the fact that despite the increased activity of enzymes that metabolize free PUFAs, such as COXs and LOX, the level of free fatty acids is significantly elevated in the plasma of patients with COVID-19. A similar direction of changes in the activity of these enzymes was observed in other studies in COVID-19 patients [42].

The group of lipid mediators that are formed during the enzymatic metabolism of arachidonic acid is eicosanoids [43], whose levels are increased in the plasma of patients with COVID-19. The increase in eicosanoids levels may be the result of increased hydrolysis of arachidonic acid-containing phospholipids, leading to an increase in free arachidonic acid, which is then a substrate for COXs and LOXs (Supplementary Figure S1), whose activity was significantly increased in COVID-19 patients. It is known that viral infections can induce COX2 at the level of mRNA and protein expression [44]. 

One of the products of COX-2 action are prostaglandins, especially PGE2, whose elevated levels can lead to chronic inflammation through the cascading release of pro-inflammatory cytokines [45], as well as the activation of pro-inflammatory T cells, mainly TH1 and TH17 [46]. In addition, during chronic inflammation, the recruitment of immune cells (e.g., macrophages, T lymphocytes, and B lymphocytes) was observed through synergistic interaction with chemokines [47] as well as an increase in pro-inflammatory genes induced by cytokines [18]. Therefore, significantly elevated PGE2 levels may be one of the main factors leading to the intensification of COVID-19 infection. Moreover, animal studies have shown that PGE2 levels depend on age [48], which, in the context of the average age of COVID-19 patients (61/72) participating in this study, may be very important. Although PGE2 is considered a pro-inflammatory, mediator there are also reports indicating its anti-inflammatory and regenerative effects [49,50]. PGE2 may participate in the conversion of pro-inflammatory interleukins (e.g., IL-1β and IL-6) to anti-inflammatory interleukins synthesized by M2 macrophages (e.g., IL-10) [51], which may explain the significant increase in IL-10 in patients who survived COVID-19. It is suggested that this is related to the transformation of pro-inflammatory M1 macrophages into anti-inflammatory M2 by increasing the activity of the enzyme 15-LOX, which is primarily involved in the synthesis of pro-resolving lipid mediators [52]. It has also been suggested that PGE2 levels are elevated in the early stages of inflammation, as we observed in our study because the blood used for this study was drawn from patients admitted to the hospital. 

The results of this study also show increased levels of thromboxane B2 (TXB2), especially in the plasma of patients who died after 7 days. A similar direction of change was also detected in critically ill patients due to COVID-19, leading to the suggestion of a correlation between increased TXB2 generation and thrombosis and mortality among patients [53,54]. This is clearly confirmed by the fact that the derivative of TXB2—11-dehydro-thromboxane B2 (11-dh-TXB2) is responsible for thrombotic events and higher mortality, and therefore is even indicated as a potential prognostic biomarker for COVID-19 patients [55]. 

In contrast, increased levels of anti-inflammatory eicosanoids (15-d-PGJ2 and 12-HETE) were observed in COVID-19 survivors. This situation may be related to the fact that 15-d-PGJ2 is an agonist of PPAR-γ receptors, which exert anti-inflammatory effects by the inactivation of NFκB. Mechanisms of inactivation include direct binding and thus inactivation of p65 NFκB or ubiquitination of this protein, leading to its proteolytic degradation. Moreover, it is known that 15-d-PGJ2 may decrease genes responsible for the biosynthesis of proinflammatory proteins, including COX2, TNFα, and IL-6 [56,57]. This may result in a smaller increase in the activity of COX2 and TNFα expression than would be expected in plasma from COVID-19 patients. Moreover, the PPAR-γ receptor, activated by 15-d-PGJ2 also cooperates with the transcription factor Nrf2 responsible for the biosynthesis of antioxidant proteins The above interactions are responsible for maintaining high expression of both the transcription factor and target antioxidant genes [58], which may support the body’s defense response. This corresponds to changes in the expression of Nrf2 and antioxidant proteins in the granulocytes of the same COVID-19 patients, presented in the work sent to the editor (Zarkovic, 2022—Cells). Thus, upregulation of 15-d-PGJ2 level may be a prognostic factor indicating the body’s ability to counteract oxidative stress and inflammation and consequently increase the likelihood of survival of COVID-19 infection, and drugs that are agonists of PPARγ receptors may be suggested as components of the pharmacotherapy of this disease.

Another eicosanoid whose level was increased in the plasma of COVID-19 patients, especially survivors, was 12-HETE. Although the intracellular generation of 12-HETE promotes increased oxidative stress, when it is localized extracellularly, this eicosanoid affects various signaling pathways, leading to activation of ERK1/2, MEK, and NFκB [59,60]. Moreover, it was shown that in the bronchoalveolar lavage fluids of COVID-19 patients, levels of 12-HETE and 15-HETE were significantly elevated compared to healthy subjects [61]. At the same time, however, it was found that 12-HETE weakens the replication of the PR8 virus in the bodies of the mice [62], which may also apply to SARS-CoV-2.

In summary, it can be concluded that oxidative stress accompanying COVID-19 promotes enhanced both ROS- and enzyme-dependent lipid metabolism. However, lipid metabolism can also be directly modified by pro-inflammatory cytokines, the levels of which can be altered by interactions of lipid mediators with G-protein coupled receptors [63]. In general, an increase in cytokine levels is observed in the second week after the onset of disease symptoms [64], which may significantly worsen the patient’s condition by inducing systemic inflammation, leading to multi-organ failure [64]. This could have resulted in the death of some patients whose metabolic changes were analyzed in this study. It is known that one of the pro-inflammatory cytokines, the levels of which are elevated especially in acute lung injury and facilitate the interaction of SARS-CoV-2 with the angiotensin-converting enzyme 2, is TNFα. This leads to the overproduction of angiotensin II as well as increased pulmonary vascular permeability and lung damage [65]. It is further suggested that IL-10 may be a better marker of the disease conditions because its levels are elevated earlier than IL-6 in patients with COVID-19 as indicated earlier [66]. Consequently, the changes observed in patients admitted to the hospital may not yet correspond to the final metabolic response accompanying COVID-19. The more severe symptoms in recovered patients than in patients who died may be related more to the period of development of the disease and not correspond to the final state. Reduced metabolic responses of the patients who died may also indicate the body’s inability to respond adequately to the presence of the pathogen in the body. It should also be taken into account that both lipid and protein mediators (cytokines), depending on environmental conditions, may have different functions, including both inhibiting and promoting inflammation and the body’s innate and adaptive immune responses. Thus, a stronger increase in pro-inflammatory factors in recovered patients than in deceased patients may be the result of the body’s defensive response, which may also be a prognostic factor. On the other hand, observations regarding changes at the level of lipid mediators may suggest their modulation as part of the pharmacotherapy of seriously ill patients.

## 4. Materials and Methods

### 4.1. Samples Collection

The following blood samples were taken from patients with COVID-19: 66 who recovered (25 female and 41 men), mean age: 65 (53–72) and 22 patients with COVID-19 who died, (13 female and 9 men), mean age 72 (66–81) treated in the Clinical Hospital Dubrava in Zagreb, serving as the national COVID-19 center, thus providing medical care caring for patients suffering from the most aggressive COVID-19. The patients were treated at the Dubrava Clinical Hospital in Zagreb (Croatia), acting as the national COVID-19 center, thus providing medical care to patients suffering from the most aggressive COVID-19. Following the blood collection, patients were treated according to standard COVID-19 treatment guidelines and an individual assessment of the severity of each patient’s disease based on the need for respiratory support to account for up to 93% of capillary blood oxygen saturation. Severe illness was defined as the need for oxygen supplementation during spontaneous breathing or high-flow oxygenation or mechanical ventilation to achieve a specific goal (8 L/min). Spontaneously breathing patients who needed less than 8 L/min of oxygen were selected as moderate patients. However, differences in treatment of patients with severe or moderate disease, as well as individual differences between them, did not affect the results of the study, since patients’ blood was drawn upon admission to the hospital, i.e., before any treatment was applied.

The control group consisted of 33 healthy donors (24 female and 9 men, mean age: 45 (34–61) (Table 2). This study was conducted after obtaining the ethical approval 2020-1012-13 of the Clinical Hospital Dubrava in Zagreb.

Blood samples were drawn by venepuncture and collected into ethylenediaminetetraacetic acid (EDTA) tubes with BHT and were centrifuged at 3000× *g* (4 °C) for 20 min to obtain plasma. Plasma samples were stored at −80 °C for subsequent analysis. 

### 4.2. Methods

#### 4.2.1. Determination of Phospholipid Metabolism

The phospholipid and free fatty acids were analyzed by gas chromatography [67]. Fatty acids were isolated by Folch extraction using chloroform/methanol mixture (2:1, *v*/*v*) in the presence of 0.01% butylated hydroxytoluene. Using TLC analytes were separated with the mobile phase as follows: heptane-diisopropyl ether–acetic acid (60:40:3, *v*/*v*/*v*). All lipid fractions were transmetylated to fatty acid methyl esters (FAMEs) with boron trifluoride in methanol. FAMEs were analyzed by gas chromatography with a flame ionization detector (FID) on Clarus 500 Gas Chromatograph (Perkin Elmer). Separation of FAMEs was carried out on capillary column coated with Varian CP-Sil88 stationary phase (50 m × 0.25 mm, ID 0.2 μm, Varian). Identification of FAMEs was made by comparison of their retention time with standards and quantitation was achieved using an internal standard method (nonadecanoic acid (19:0) and 1,2-dinonadecanoyl-*sn*-glycero-3-phosphocholine (19:0 PC) were used as internal standards). Plasma levels of PL-AA, PL-DHA, free AA, and free DHA, were expressed in μg/mL. 

The activity of enzymes involved in the metabolism of phospholipids was examined spectrophotometrically in accordance with the manufacturer’s instructions as follows: phospholipase A2 (PLA2–EC.3.1.1.4) using PLA2 Assay Kit (Cayman Chemical Company, Ann Arbor, MI, USA), cyclooxygenases 1 and 2 (COX-1/2–EC.1.14.99.1) using a commercial assay kit (Cayman Chemical Company, Ann Arbor, MI, USA), lipoxygenase (LOX) using a commercial assay kit (Sigma-Aldrich, Steinheim, Germany). To detect PLA2 activity arachidonoyl thio-PC was used as a substrate. Hydrolysis of the arachidonoyl thioester bond at the sn-2 position by PLA2 was released a free thiol which was detected spectrophotometrically by reaction with (5,5’-dithio-bis(2-nitrobenzoic acid) (DTNB) at 405 nm [68]. One unit (U) of enzyme hydrolyzes one µmol of arachidonoyl Thio-PC per minute at 25 °C. COX 1 and 2 activity was measured spectrophotometrically (at 590 nm) by monitoring the appearance of oxidized N,N,N’,N’-tetramethyl-p-phenylenediamine (TMPD) [69] while measuring only COX-2 activity, the specific COX-1 SC-560 inhibitor included in the kit was used. Cyclooxygenases activities were expressed in nmol/min/mL. The increase in fluorescent signal was measured at λ_Ex_ = 500 nm and λ_Em_ = 536 nm, which was directly proportional to LOX activity. One unit (U) of LOX was determined as the amount of enzyme that causes oxidation of 1 µmol of the LOX probe per minute at pH 7.4 and at room temperature. LOX activity was expressed in mU/mL.

#### 4.2.2. Determination of the Level of Lipid Peroxidation Products 

Lipid peroxidation in plasma was estimated by measuring small molecular weight reactive aldehyde, malondialdehyde (MDA) as well as F2-isoprostanes (8-isoPGF2α). The reactive aldehyde was determined using gas chromatography coupled with mass spectrometry 7890A GC–7000 (Agilent Technologies, Palo Alto, CA, USA) as the O-pentafluorobenzyl-oxime (O-PFB-oxime) or O-pentafluorobenzyl-oxime-trimethyl silane (O-PFB-oxime-TMS) derivatives, based on Luo’s method [70]. Benzaldehyde-d6 was added to plasma as an internal standard. Aldehyde derivatives were separated using an HP- 5 ms capillary column (0.25-mm internal diameter, 0.25-μm film thickness, 30-m length) and analyzed in selected ion monitoring mode (SIM). Samples were deproteinized by the addition of 1 mL of methanol. MDA derivatives were extracted with hexane. The hexane layer was evaporated and N,O-bis (trimethylsilyl) trifluoroacetamide in 1% trimethylchlorosilane was added. The following ions were monitored: *m*/*z* 204.0 and 178.0 for MDA-PFB and *m*/*z* 307.0 for IS (benzaldehyde-D_6_) derivatives. Plasma level of MDA was expressed in nmol/mL. 

Total isoprostanes (measured as 8-isoPGF2α) level was quantified using modified LC-MS methods of Coolen [71]. Analysis by LC-MS was conducted on a Shimadzu (Kyoto, Japan) Triple Quad 8060 Mass Spectrometer and a Shimadzu UHPLC System, while the liquid chromatographic separation was acquired via Eclipse Plus C18 analytical column (2.1 × 100 mm, 1.8 µm particle size). Briefly, 8-isoPGF2α in plasma samples was extracted, purified, and quantified using the stable isotope dilution LC-MS technique. The 8-isoPGF2α–d4 as an internal standard was used. The 8-isoPGF2α was analyzed in negative ion mode using MRM. Transitions of the precursor to the production as follows: *m/z* 353.2→193.1 and 357.2→197.1 were used for 8-isoPGF2α and 8-isoPGF2α-d4 respectively. Plasma level of 8-isoPGF2α was expressed in ng/mL. 

#### 4.2.3. Determination of the Level of Lipids Mediators (Endocannabinoids and Eicosanoids) 

Endocannabinoids: AEA, 2-AG, N-oleoylethanolamine (OEA), and N-palmitoylethanolamine (PEA) were determined using ultra-performing liquid chromatography-tandem mass spectrometry (UPLC-MS/MS) [72]. Analysis by LC-MS was conducted on a Shimadzu (Kyoto, Japan) Triple Quad 8060 Mass Spectrometer and a Shimadzu UHPLC System, while the liquid chromatographic separation was acquired via Poroshell 120 EC-C18 analytical column (3.0 × 150 mm; 2.7 µm particle size). Briefly, AEA, 2-AG, OEA, and PEA were extracted from plasma samples, purified, and quantified using the stable isotope dilution LC-MS technique. Deuterated endocannabinoids: AEA-d_8_, 2-AG-d_8_, and OEA-d_4_ as internal standards were used. The samples were analyzed in positive-ion mode using multiple reaction monitoring (MRM). Transitions of the precursors to the product ions were as follows: *m/z* 348.3→62.15 for AEA, *m/z* 379.3→287.25 for 2-AG, *m/z* 300.3→62.0 for PEA, 326.3→62.0 for OEA, *m/z* 356.2→63.05 for AEA-d8, *m/z* 387.3→294.0 for 2-AG-d8 and *m/z* 330.20→66.15 for OEA-d4. Plasma levels of AEA, 2-AG, OEA, and PEA were measured against a standard curve and then expressed as pmol/mL.

Eicosanoids: thromboxane B2 (TXB_2_), prostaglandin E2 (PGE_2_), 15-deoxy-delta12,14-prostaglandin J2 (15d-PGJ_2_), and 12-hydroxyeicosatetraenoic acid (12-HETE) were determined using ultra-performing liquid chromatography-tandem mass spectrometry (UPLC-MS/MS) [73]. Analysis by LC-MS was conducted on Shimadzu (Kyoto, Japan) Triple Quad 8060 Mass Spectrometer and a Shimadzu UHPLC System, while the liquid chromatographic separation was acquired via an Eclipse Plus C18 analytical column (2.1 × 100 mm, 1.8 µm particle size). Briefly, TXB_2_, PGE_2_, 15d-PGJ_2_, and 12-HETE in plasma samples were extracted, purified, and quantified using the stable isotope dilution LC-MS technique. Deuterated eicosanoids: TXB_2_-d_4_, PGD_2_-d_4_, 15-d-PGJ_2_-d_4_, and 15-HETE-d_8_ were used as internal standards. The samples were analyzed in positive-ion mode using multiple reaction monitoring (MRM). Transitions of the precursor to the product ions were as follows: *m/z* 351.3→271.2 for PGE_2_, *m/z* 315.2→271.2 for 15-d-PGJ_2_, *m/z* 369.3→169.1 for TXB_2_, *m/z* 319.2→179.1 for 12-HETE, *m/z* 355.0→275.3 for PGD_2_-d_4_, *m/z* 373.0→173.1 for TXB_2_-d_4_ *m/z* 319.3→275.2 for 15-d-PGJ_2_-d_4_, and 327.0→226.2 for 15-HETE-d_8_. Plasma levels of TXB_2_, PGE_2_, 15d-PGJ_2_, and 12-HETE were measured against a standard curve and then expressed as pmol/mL.

#### 4.2.4. Determination of the Level of TNFα and IL-10 

Measurement of plasma protein expression was performed using enzyme-linked immunosorbent assay (ELISA) [74]. Plasma was applied to ELISA plate wells (Nunc Immuno MaxiSorp, Thermo Scientific, Waltham, MA, USA). Plates with attached proteins were incubated (4 °C) for 3h with blocking solution (5% fat-free dry milk in carbonate binding buffer). After washing with PBS supplemented with 0.1% Tween 20, plasma samples were incubated at 4^0^C overnight with appropriate primary antibodies against IL-10 (host:mouse) (Santa Cruz Biotechnology, CA, USA); TNFα (host: rabbit) (Sigma-Aldrich, St. Louis, MO, USA). Next, following washing (PBS supplemented with 0.1% Tween 20), plates were incubated for 30 min with peroxidase blocking solution (3% hydrogen peoroxide, 3% fat-free dry milk in PBS) at room temperature. As a secondary antibody goat anti-rabbit/mouse EnVision + Dual Link/HRP solution (1:100) (Agilent Technologies, Santa Clara, CA, USA) was used. After 1 h of incubation at room temperature, secondary antibody was removed and plate was incubated with chromogen substrate solution (0.1 mg/mL TMB, 0.012% H_2_O_2_) for 40 min. The reaction was stopped by adding 2 M sulfuric acid and absorption was read within 10 min at 450 nm and automatically recalculated from standard curves for IL-10 (Fine, Test Wuhan, Hubei, China) and TNFα (Merck, Darmstadt, Germany) and then expressed as ng/mL.

## Figures and Tables

**Table 1:** Comparison of some laboratory data of patients with COVID-19 in respect to normal values. *—significant (*p* < 0.05) difference to the values of recovered patients.

	Normal Range	COVID-19 Recovered	COVID-19 Deceased
WBC [10^3^/μL]	4.00–10.00	11.17 ± 3.44	11.43 ± 3.52
Neutrophils [%]	40.0–72.0	80.67 ± 5.54	86.60 ± 4.62 *
Platelets [10^3^/μL]	150–400	292.50 ± 105.14	226.61 ± 57.37
Blood oxygen saturation [%]	>95%	91.17 ± 5.65	90.67 ± 5.33
Ferritin [μg/L]	11–336	913 ± 475	943 ± 436
PCT [ng/mL]	<0.1	0.38 ± 0.35	1.04 ± 1.09
LDH [U/L]	140–280	420 ± 130	358 ± 140
CRP [mg/L]	0.00–5.00	139.01 ± 74.08	185.14 ± 59.37
IL-6 [pg/mL]	0–43.5	87 ± 45	185 ± 104 *

**Table 2:** Demographic and clinical characteristics of patients with COVID-19 compared to healthy subjects.

	Healthy Subjects	COVID-19 Recovered	COVID-19 Deceased
Age (years)	45 ± 12.6	58.9 ± 9.1 ^a^	72.3 ± 6.9 ^a,b^
Sex	24F 9M	25F 41M	13F 9M
Body Mass Index	25.9 ± 4.7	32.5 ± 5.8 ^a^	28.5 ± 3.3 ^b^

Data points represent the mean ± SD; ^a^, significantly different from healthy subject, *p* < 0.05; ^b^, significantly different from patients with COVID-19 recovered, *p* < 0.05.

## Data Availability

The data presented in this study are contained within the article.

## Supplementary Materials

The following supporting information can be downloaded at: https://www.mdpi.com/article/10.3390/ijms231911810/s1

## References

[1] Sharma A., Tiwari S., Deb M.K., Marty J.L. (2020). Severe Acute Respiratory Syndrome Coronavirus-2 (SARS-CoV-2): A Global Pandemic and Treatment Strategies. Int. J. Antimicrob. Agents.

[2] Zaim S., Chong J.H., Sankaranarayanan V., Harky A. (2020). COVID-19 and Multiorgan Response. Curr. Probl. Cardiol..

[3] De Las Heras N., Martín Giménez V.M., Ferder L., Manucha W., Lahera V. (2020). Implications of Oxidative Stress and Potential Role of Mitochondrial Dysfunction in COVID-19: Therapeutic Effects of Vitamin D. Antioxidants.

[4] Chernyak B.V., Popova E.N., Prikhodko A.S., Grebenchikov O.A., Zinovkina L.A., Zinovkin R.A. (2020). COVID-19 and Oxidative Stress. Biochemistry.

[5] Soto M.E., Guarner-Lans V., Díaz-Díaz E., Manzano-Pech L., Palacios-Chavarría A., Valdez-Vázquez R.R., Aisa-Álvarez A., Saucedo-Orozco H., Pérez-Torres I. (2022). Hyperglycemia and Loss of Redox Homeostasis in COVID-19 Patients. Cells.

[6] Žarković N., Orehovec B., Milković L., Baršić B., Tatzber F., Wonisch W., Tarle M., Kmet M., Mataić A., Jakovčević A. (2021). Preliminary Findings on the Association of the Lipid Peroxidation Product 4-Hydroxynonenal with the Lethal Outcome of Aggressive COVID-19. Antioxidants.

[7] Schwarz B., Sharma L., Roberts L., Peng X., Bermejo S., Leighton I., Casanovas-Massana A., Minasyan M., Farhadian S., Ko A.I. (2021). Cutting Edge: Severe SARS-CoV-2 Infection in Humans Is Defined by a Shift in the Serum Lipidome, Resulting in Dysregulation of Eicosanoid Immune Mediators. J. Immunol..

[8] Martín-Fernández M., Aller R., Heredia-Rodríguez M., Gómez-Sánchez E., Martínez-Paz P., Gonzalo-Benito H., Sánchez-de Prada L., Gorgojo Ó., Carnicero-Frutos I., Tamayo E. (2021). Lipid Peroxidation as a Hallmark of Severity in COVID-19 Patients. Redox Biol..

[9] Zarkovic N., Jakovcevic A., Mataic A., Jaganjac M., Vukovic T., Waeg G., Zarkovic K. (2022). Post-Mortem Findings of Inflammatory Cells and the Association of 4-Hydroxynonenal with Systemic Vascular and Oxidative Stress in Lethal COVID-19. Cells.

[10] Nagoor Meeran M.F., Sharma C., Goyal S.N., Kumar S., Ojha S. (2021). CB2 Receptor-Selective Agonists as Candidates for Targeting Infection, Inflammation, and Immunity in SARS-CoV-2 Infections. Drug Dev. Res..

[11] Cinar R., Iyer M.R., Kunos G. (2022). Dual Inhibition of CB1 Receptors and INOS, as a Potential Novel Approach to the Pharmacological Management of Acute and Long COVID-19. Br. J. Pharmacol..

[12] Sultan M., Alghetaa H., Mohammed A., Abdulla O.A., Wisniewski P.J., Singh N., Nagarkatti P., Nagarkatti M. (2021). The Endocannabinoid Anandamide Attenuates Acute Respiratory Distress Syndrome by Downregulating MiRNA That Target Inflammatory Pathways. Front. Pharmacol..

[13] Snider J.M., You J.K., Wang X., Snider A.J., Hallmark B., Zec M.M., Seeds M.C., Sergeant S., Johnstone L., Wang Q. (2021). Group IIA Secreted Phospholipase A_2_ Is Associated with the Pathobiology Leading to COVID-19 Mortality. J. Clin. Investig..

[14] Palmas F., Clarke J., Colas R.A., Gomez E.A., Keogh A., Boylan M., McEvoy N., McElvaney O.J., McElvaney O., Alalqam R. (2021). Dysregulated Plasma Lipid Mediator Profiles in Critically Ill COVID-19 Patients. PLoS ONE.

[15] Dalli J., Colas R.A., Serhan C.N. (2013). Novel N-3 Immunoresolvents: Structures and Actions. Sci. Rep..

[16] Pistorius K., Souza P.R., De Matteis R., Austin-Williams S., Primdahl K.G., Vik A., Mazzacuva F., Colas R.A., Marques R.M., Hansen T.V. (2018). PDn-3 DPA Pathway Regulates Human Monocyte Differentiation and Macrophage Function. Cell Chem. Biol..

[17] Serhan C.N., Chiang N., Dalli J. (2018). New Pro-Resolving n-3 Mediators Bridge Resolution of Infectious Inflammation to Tissue Regeneration. Mol. Asp. Med..

[18] Dennis E.A., Norris P.C. (2015). Eicosanoid Storm in Infection and Inflammation. Nat. Rev. Immunol..

[19] Sahanic S., Löffler-Ragg J., Tymoszuk P., Hilbe R., Demetz E., Masanetz R.K., Theurl M., Holfeld J., Gollmann-Tepeköylü C., Tzankov A. (2021). The Role of Innate Immunity and Bioactive Lipid Mediators in COVID-19 and Influenza. Front. Physiol.

[20] Vijay R., Hua X., Meyerholz D.K., Miki Y., Yamamoto K., Gelb M., Murakami M., Perlman S. (2015). Critical Role of Phospholipase A2 Group IID in Age-Related Susceptibility to Severe Acute Respiratory Syndrome-CoV Infection. J. Exp. Med..

[21] Rajasagi N.K., Reddy P.B.J., Mulik S., Gjorstrup P., Rouse B.T. (2013). Neuroprotectin D1 Reduces the Severity of Herpes Simplex Virus-Induced Corneal Immunopathology. Investig. Ophthalmol. Vis. Sci..

[22] Koenis D.S., Beegun I., Jouvene C.C., Aguirre G.A., Souza P.R., Gonzalez-Nunez M., Ly L., Pistorius K., Kocher H.M., Ricketts W. (2021). Disrupted Resolution Mechanisms Favor Altered Phagocyte Responses in COVID-19. Circ. Res..

[23] McElvaney O.J., McEvoy N.L., McElvaney O.F., Carroll T.P., Murphy M.P., Dunlea D.M., Ní Choileáin O., Clarke J., O’Connor E., Hogan G. (2020). Characterization of the Inflammatory Response to Severe COVID-19 Illness. Am. J. Respir. Crit. Care Med..

[24] Kessler B., Rinchai D., Kewcharoenwong C., Nithichanon A., Biggart R., Hawrylowicz C.M., Bancroft G.J., Lertmemongkolchai G. (2017). Interleukin 10 Inhibits Pro-Inflammatory Cytokine Responses and Killing of *Burkholderia Pseudomallei*. Sci. Rep..

[25] Hammock B.D., Wang W., Gilligan M.M., Panigrahy D. (2020). Eicosanoids: The Overlooked Storm in Coronavirus Disease 2019 (COVID-19)?. Am. J. Pathol..

[26] Casari I., Manfredi M., Metharom P., Falasca M. (2021). Dissecting Lipid Metabolism Alterations in SARS-CoV-2. Prog. Lipid Res..

[27] Gallo C.G., Fiorino S., Posabella G., Antonacci D., Tropeano A., Pausini E., Pausini C., Guarniero T., Hong W., Giampieri E. (2022). The Function of Specialized Pro-Resolving Endogenous Lipid Mediators, Vitamins, and Other Micronutrients in the Control of the Inflammatory Processes: Possible Role in Patients with SARS-CoV-2 Related Infection. Prostaglandins Other Lipid Mediat..

[28] Mas-Bargues C., Escrivá C., Dromant M., Borrás C., Viña J. (2021). Lipid Peroxidation as Measured by Chromatographic Determination of Malondialdehyde. Human Plasma Reference Values in Health and Disease. Arch. Biochem. Biophys..

[29] Komaravelli N., Casola A. (2014). Respiratory Viral Infections and Subversion of Cellular Antioxidant Defenses. J. Pharm. Pharm..

[30] Finn D.P., Haroutounian S., Hohmann A.G., Krane E., Soliman N., Rice A.S.C. (2021). Cannabinoids, the Endocannabinoid System, and Pain: A Review of Preclinical Studies. Pain.

[31] Rahman S.M.K., Uyama T., Hussain Z., Ueda N. (2021). Roles of Endocannabinoids and Endocannabinoid-Like Molecules in Energy Homeostasis and Metabolic Regulation: A Nutritional Perspective. Annu. Rev. Nutr..

[32] Sultan M., Wilson K., Abdulla O.A., Busbee P.B., Hall A., Carter T., Singh N., Chatterjee S., Nagarkatti P., Nagarkatti M. (2021). Endocannabinoid Anandamide Attenuates Acute Respiratory Distress Syndrome through Modulation of Microbiome in the Gut-Lung Axis. Cells.

[33] Gubernatorova E.O., Gorshkova E.A., Polinova A.I., Drutskaya M.S. (2020). IL-6: Relevance for Immunopathology of SARS-CoV-2. Cytokine Growth Factor Rev..

[34] McGonagle D., Sharif K., O’Regan A., Bridgewood C. (2020). The Role of Cytokines Including Interleukin-6 in COVID-19 Induced Pneumonia and Macrophage Activation Syndrome-Like Disease. Autoimmun. Rev..

[35] Hernández-Cervantes R., Méndez-Díaz M., Prospéro-García Ó., Morales-Montor J. (2017). Immunoregulatory Role of Cannabinoids during Infectious Disease. NIM.

[36] Krishnan G., Chatterjee N. (2014). Endocannabinoids Affect Innate Immunity of Muller Glia during HIV-1 Tat Cytotoxicity. Mol. Cell. Neurosci..

[37] Mestre L., Iñigo P.M., Mecha M., Correa F.G., Hernangómez-Herrero M., Loría F., Docagne F., Borrell J., Guaza C. (2011). Anandamide Inhibits Theiler’s Virus Induced VCAM-1 in Brain Endothelial Cells and Reduces Leukocyte Transmigration in a Model of Blood Brain Barrier by Activation of CB1receptors. J. Neuroinflamm..

[38] Turcotte C., Blanchet M.-R., Laviolette M., Flamand N. (2016). The CB2 Receptor and Its Role as a Regulator of Inflammation. Cell. Mol. Life Sci..

[39] Ghaffari S., Roshanravan N., Tutunchi H., Ostadrahimi A., Pouraghaei M., Kafil B. (2020). Oleoylethanolamide, A Bioactive Lipid Amide, as A Promising Treatment Strategy for Coronavirus/COVID-19. Arch. Med. Res..

[40] Schönrich G., Raftery M.J., Samstag Y. (2020). Devilishly Radical NETwork in COVID-19: Oxidative Stress, Neutrophil Extracellular Traps (NETs), and T Cell Suppression. Adv. Biol. Regul..

[41] Jensen M.D., Sheng W., Simonyi A., Johnson G.S., Sun A.Y., Sun G.Y. (2009). Involvement of Oxidative Pathways in Cytokine-Induced Secretory Phospholipase A2-IIA in Astrocytes. Neurochem. Int..

[42] Mazidimoradi A., Alemzadeh E., Alemzadeh E., Salehiniya H. (2022). The Effect of Polyunsaturated Fatty Acids on the Severity and Mortality of COVID Patients: A Systematic Review. Life Sci..

[43] Biagini D., Franzini M., Oliveri P., Lomonaco T., Ghimenti S., Bonini A., Vivaldi F., Macera L., Balas L., Durand T. (2022). MS-Based Targeted Profiling of Oxylipins in COVID-19: A New Insight into Inflammation Regulation. Free Radic. Biol. Med..

[44] Savard M., Bélanger C., Tremblay M.J., Dumais N., Flamand L., Borgeat P., Gosselin J. (2000). EBV Suppresses Prostaglandin E2 Biosynthesis in Human Monocytes. J. Immunol..

[45] Honda T., Segi-Nishida E., Miyachi Y., Narumiya S. (2006). Prostacyclin-IP Signaling and Prostaglandin E2-EP2/EP4 Signaling Both Mediate Joint Inflammation in Mouse Collagen-Induced Arthritis. J. Exp. Med..

[46] Chen Q., Muramoto K., Masaaki N., Ding Y., Yang H., Mackey M., Li W., Inoue Y., Ackermann K., Shirota H. (2010). A Novel Antagonist of the Prostaglandin E(2) EP(4) Receptor Inhibits Th1 Differentiation and Th17 Expansion and Is Orally Active in Arthritis Models. Br. J. Pharmacol..

[47] Aoki T., Narumiya S. (2012). Prostaglandins and Chronic Inflammation. Trends Pharmacol. Sci..

[48] Wu D., Mura C., Beharka A.A., Han S.N., Paulson K.E., Hwang D., Meydani S.N. (1998). Age-Associated Increase in PGE2 Synthesis and COX Activity in Murine Macrophages Is Reversed by Vitamin E. Am. J. Physiol..

[49] Duffin R., O’Connor R.A., Crittenden S., Forster T., Yu C., Zheng X., Smyth D., Robb C.T., Rossi F., Skouras C. (2016). Prostaglandin E_2_ Constrains Systemic Inflammation through an Innate Lymphoid Cell-IL-22 Axis. Science.

[50] FitzGerald G.A. (2015). BIOMEDICINE. Bringing PGE_2_ in from the Cold. Science.

[51] Serhan C.N., Chiang N., Van Dyke T.E. (2008). Resolving Inflammation: Dual Anti-Inflammatory and pro-Resolution Lipid Mediators. Nat. Rev. Immunol..

[52] Elliott M.R., Koster K.M., Murphy P.S. (2017). Efferocytosis Signaling in the Regulation of Macrophage Inflammatory Responses. J. Immunol..

[53] Canzano P., Brambilla M., Porro B., Cosentino N., Tortorici E., Vicini S., Poggio P., Cascella A., Pengo M.F., Veglia F. (2021). Platelet and Endothelial Activation as Potential Mechanisms Behind the Thrombotic Complications of COVID-19 Patients. JACC Basic Transl. Sci..

[54] Perico L., Benigni A., Casiraghi F., Ng L.F.P., Renia L., Remuzzi G. (2021). Immunity, Endothelial Injury and Complement-Induced Coagulopathy in COVID-19. Nat. Rev. Nephrol..

[55] Tantry U.S., Bliden K.P., Cho A., Walia N., Dahlen J.R., Ens G., Traianova M., Jerjian C., Usman A., Gurbel P.A. (2021). First Experience Addressing the Prognostic Utility of Novel Urinary Biomarkers in Patients With COVID-19. Open Forum Infect. Dis..

[56] Korbecki J., Bobiński R., Dutka M. (2019). Self-Regulation of the Inflammatory Response by Peroxisome Proliferator-Activated Receptors. Inflamm. Res..

[57] Schwager J., Gagno L., Richard N., Simon W., Weber P., Bendik I. (2018). Z-Ligustilide and Anti-Inflammatory Prostaglandins Have Common Biological Properties in Macrophages and Leukocytes. Nutr. Metab..

[58] Lee C. (2017). Collaborative Power of Nrf2 and PPARγ Activators against Metabolic and Drug-Induced Oxidative Injury. Oxid. Med. Cell. Longev..

[59] Guo Y., Zhang W., Giroux C., Cai Y., Ekambaram P., Dilly A.-K., Hsu A., Zhou S., Maddipati K.R., Liu J. (2011). Identification of the Orphan G Protein-Coupled Receptor GPR31 as a Receptor for 12-(S)-Hydroxyeicosatetraenoic Acid. J. Biol. Chem..

[60] Porro B., Songia P., Squellerio I., Tremoli E., Cavalca V. (2014). Analysis, Physiological and Clinical Significance of 12-HETE: A Neglected Platelet-Derived 12-Lipoxygenase Product. J. Chromatogr. B Analyt. Technol. Biomed. Life Sci..

[61] Archambault A.-S., Zaid Y., Rakotoarivelo V., Doré É., Dubuc I., Martin C., Amar Y., Cheikh A., Fares H., Hassani A.E. (2020). Lipid Storm within the Lungs of Severe COVID-19 Patients: Extensive Levels of Cyclooxygenase and Lipoxygenase-Derived Inflammatory Metabolites. medRxiv.

[62] Morita M., Kuba K., Ichikawa A., Nakayama M., Katahira J., Iwamoto R., Watanebe T., Sakabe S., Daidoji T., Nakamura S. (2013). The Lipid Mediator Protectin D1 Inhibits Influenza Virus Replication and Improves Severe Influenza. Cell.

[63] Torrinhas R.S., Calder P.C., Lemos G.O., Waitzberg D.L. (2021). Parenteral Fish Oil: An Adjuvant Pharmacotherapy for Coronavirus Disease 2019?. Nutrition.

[64] Weill P., Plissonneau C., Legrand P., Rioux V., Thibault R. (2020). May Omega-3 Fatty Acid Dietary Supplementation Help Reduce Severe Complications in Covid-19 Patients?. Biochimie.

[65] Guo Y., Hu K., Li Y., Lu C., Ling K., Cai C., Wang W., Ye D. (2022). Targeting TNF-α for COVID-19: Recent Advanced and Controversies. Front. Public Health.

[66] Zhao Y., Qin L., Zhang P., Li K., Liang L., Sun J., Xu B., Dai Y., Li X., Zhang C. (2020). Longitudinal COVID-19 Profiling Associates IL-1RA and IL-10 with Disease Severity and RANTES with Mild Disease. JCI Insight.

[67] Christie W.W., Esterification B.A. (1993). Preparation of Ester Derivatives of Fatty Acids for Chromatographic Analysis. Advances in Lipid Methodology-Two.

[68] Reynolds L.J., Hughes L.L., Yu L., Dennis E.A. (1994). 1-Hexadecyl-2-Arachidonoylthio-2-Deoxy-Sn-Glycero-3-Phosphorylcholine as a Substrate for the Microtiterplate Assay of Human Cytosolic Phospholipase A2. Anal. Biochem..

[69] Kulmacz R.J., Wang L.-H. (1995). Comparison of Hydroperoxide Initiator Requirements for the Cyclooxygenase Activities of Prostaglandin H Synthase-1 and −2 (∗). J. Biol. Chem..

[70] Luo X.P., Yazdanpanah M., Bhooi N., Lehotay D.C. (1995). Determination of Aldehydes and Other Lipid Peroxidation Products in Biological Samples by Gas Chromatography-Mass Spectrometry. Anal. Biochem..

[71] Coolen S.A.J., van Buuren B., Duchateau G., Upritchard J., Verhagen H. (2005). Kinetics of Biomarkers: Biological and Technical Validity of Isoprostanes in Plasma. Amino Acids.

[72] Luque-Córdoba D., Calderón-Santiago M., Luque de Castro M.D., Priego-Capote F. (2018). Study of Sample Preparation for Determination of Endocannabinoids and Analogous Compounds in Human Serum by LC-MS/MS in MRM Mode. Talanta.

[73] Watkins B.A., Kim J., Kenny A., Pedersen T.L., Pappan K.L., Newman J.W. (2016). Circulating Levels of Endocannabinoids and Oxylipins Altered by Dietary Lipids in Older Women Are Likely Associated with Previously Identified Gene Targets. Biochim. Biophys. Acta.

[74] Hnasko R., Lin A., McGarvey J.A., Stanker L.H. (2011). A Rapid Method to Improve Protein Detection by Indirect ELISA. Biochem. Biophys. Res. Commun..

