# Supplementary material for: Diversified Effects of COVID-19 as a Consequence of the Differential Metabolism of Phospholipids and Lipid Peroxidation Evaluated in the Plasma of Survivors and Deceased Patients upon Admission to the Hospital
Source: Int J Mol Sci. 2022 Oct 5;23(19):11810. doi: 10.3390/ijms231911810 (PMC9570244; doi:10.3390/ijms231911810)
Supplement: Supplementary file 1 [file ijms-23-11810-s001.zip › ijms-1930425-supplementary.pdf]

Table S1. Changes in examined parameters comparing the control to deceased COVID 19 patients and recovered COVID 19 patients group of plasma samples. One-way ANOVA analysis parameters; mean difference was significant at the 0.05 level ($p < 0.0001$****; $p < 0.001$***; $p < 0.01$**; $p < 0.05$*; n.s. – not significant).

	**Control**			**Covid 19 recovered**			**Covid 19 deceased**			**Control vs. Covid 19 recovered**	**Control vs. Covid 19 deceased**	**Covid 19 recovered vs. Covid 19 deceased**
	**Mean**	**SD**	**n**	**Mean**	**SD**	**n**	**Mean**	**SD**	**n**			
**PL-AA**	209.8	36.4	33	103.9	19.3	66	174.5	38.6	22	****	****	****
**PL-DHA**	98.8	29.3	33	50.3	12.8	66	50.1	11.8	22	****	****	ns
**free AA**	3.58	0.88	33	13.1	2.92	66	7.12	1.65	22	****	****	****
**free DHA**	2.75	0.54	33	4.11	1.03	66	3.96	0.67	22	****	****	ns
**PLA2**	10.69	2.32	33	27.59	7.81	66	13.22	3.86	22	****	ns	****
**LOX**	0.18	0.03	33	0.38	0.08	66	0.35	0.07	22	****	****	ns
**COX-1**	14.6	2.9	33	20.5	5.8	66	16.8	4.1	22	****	ns	**
**COX-2**	10.6	2.1	33	21.15	6.7	66	9.7	3.1	22	****	ns	****
**MDA**	1.61	0.38	33	2.92	0.75	66	3.52	1.05	22	****	****	**
**8-isoPGF2α**	0.99	0.34	33	4.88	1.39	66	3.87	1.15	22	****	****	**
**PGE2**	2.45	0.38	33	7.34	1.87	66	2.59	0.78	22	****	ns	****
**TXB2**	0.57	0.11	33	2.9	0.7	66	5.17	1.85	22	****	****	****
**12-HETE**	22.78	8.94	33	99.66	31.2	66	34.33	8.98	22	****	ns	****
**15-d-PGJ2**	10.09	2.46	33	18.34	4.76	66	11.17	3.08	22	****	ns	****
**2-AG**	4.04	0.77	33	11.25	2.56	66	7.05	1.43	22	****	****	****
**AEA**	2.34	0.52	33	5.59	1.25	66	5.57	1.54	22	****	****	ns
**PEA**	25.98	5.3	33	37.67	7.8	66	41.53	9.2	22	***	****	ns
**OEA**	11.02	2.32	33	15.63	4.22	66	18.52	4.56	22	****	****	**
**TNFα**	7.1	1.05	33	11.13	2.64	66	8.94	1.27	22	****	**	***
**IL-10**	0.64	0.13	33	1.01	0.17	66	0.76	0.1	22	****	*	****

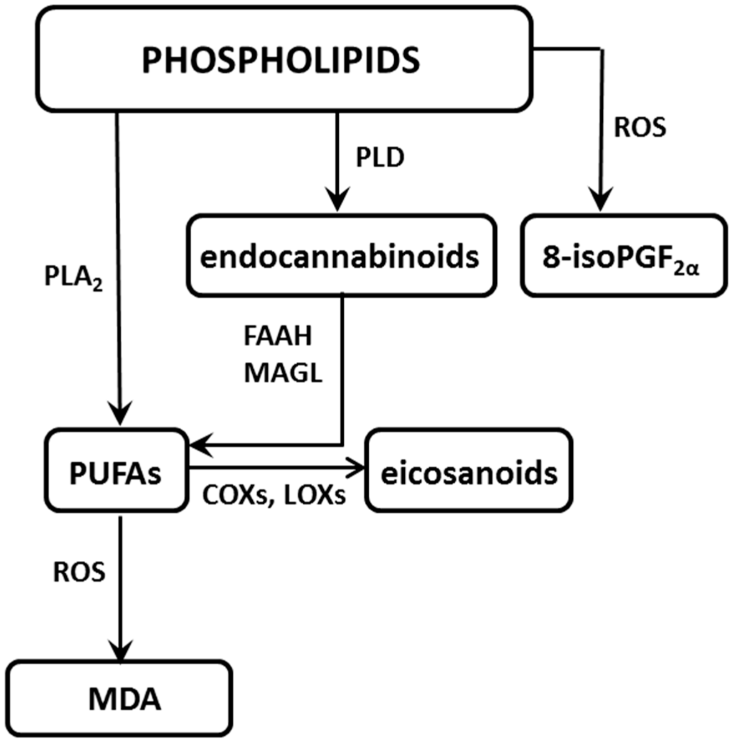

Figure S1. Phospholipid metabolism in response to reactions with reactive oxygen species (ROS) and enzymes (cyclooxygenases-(COXs) and lipoxygenases-(LOXs)).

FAAH - fatty acid amide hydrolase, MAGL - monoacylglycerol lipase, MDA - malondialdehyde, PLA2 – phospholipase A_2, PLD - phospholipase D, PUFAs – polyunsaturated fatty acids.
